# A case–control study of filicide/infanticide in 90 mothers

**DOI:** 10.1007/s00737-023-01401-5

**Published:** 2023-12-13

**Authors:** Alessandra Bramante, Arianna Di Florio

**Affiliations:** 1 Società Italiana Per La Salute Mentale Perinatale, Milan, Italy; 2 Division of Psychological Medicine and Clinical Neurosciences, Cardiff University, Hadyn Ellis Building, Room 2.12, Maindy Road, Cardiff, CF24 4HQ UK

**Keywords:** Filicide, Infanticide, Postpartum psychosis, Schizophrenia, Bipolar disorder

## Abstract

This study aims to explore the clinical and socio-demographic characteristics of 30 women who committed filicide and compare them to those of 60 postpartum women who did not commit filicide, including 30 with severe postpartum mental illness and 30 without a known history of psychiatric disorders. Clinical assessment included a face-to-face interview with the Structured Clinical Interviews for DSM-IV Axis I and Axis II Disorders. Information on socio-economic, medical, and personal factors was collected using the Clinical Interview for DSM-IV and organized in a clinical vignette and OPCRIT ratings. Consensus best-estimate diagnoses were made according to DSM-V criteria. Inference was conducted using Fisher’s exact test for categorical variables and Mann–Whitney *U* rank test for continuous variables. Family history of violent death, psychotic symptoms (OR 8.3; CI 95% 2.26–36.13), severe insomnia (9.8; 2.28–61.75), and a schizophrenia spectrum or bipolar diathesis (4.8; 1.22–23.86) were associated with history of filicide. Rates of history of sexual abuse in childhood were higher in both the filicide and the severe postpartum mental illness groups compared to healthy controls (6.7; 1.25–70.46 and 7.8; 1.47; 80.47 respectively). Conversely, we did not observe any difference in the rates of history of sexual abuse in adulthood across groups. The lack of adequate postpartum psychiatric care was an important precipitating factor in many cases of infanticide and even late filicide. This study underscores the need for increasing awareness by health care professionals and the wider society of the complex dynamics and psychiatric risks associated with motherhood.

## Introduction

There is robust evidence of an association between maternal filicide, i.e., the murder of a child by the mother and psychiatric disorders (Alder and Polk 2001; Crimmins et al. 1997; Friedman et al. 2005a; Mckee 2006; Moodley et al. 2019; Putkonen et al. 2011).

Most of the research, however, has been conducted in a judicial framework or has been limited to case reports. Only a few studies have systematically investigated the psychopathology and risk factors associated with maternal filicide. The few systematic studies have often been limited to case note reviews, without structured interviews and without systematic face-to-face assessment of women (Giacco et al. 2023).

In this case–control study, we explored the clinical and socio-demographic characteristics of 30 women who committed filicide and compared them to those of 60 postpartum women who did not commit filicide, including 30 with severe postpartum mental illness and 30 without known history of psychiatric disorders.

Compared to previous research, the ascertainment of women who committed filicide went beyond the judicial system to include clinical and rehabilitation centers. Moreover, the assessment integrated the review of clinical and judicial records with structured interviews and validated questionnaires. To our knowledge, this is also the first study on filicide using two control groups: women with postpartum mental illness and healthy postpartum women.

## Material and methods

In the original study conducted by AB, potential participants with a history of filicide (*N* = 33, thereafter *filicide group*) were systematically identified in the only Italian forensic psychiatric hospital (*N* = 19), five detention centers (*N* = 5), and five community psychiatric centers (*N* = 9) from 2009 to 2012. Permission to approach the participant was initially sought from the directors of the centers. Permission was denied for three women, two detainees, and one living in a community psychiatric center. All the remaining 30 women were then approached and accepted to take part in the study by providing written informed consent.

The terms neonaticide and infanticide will be used thereafter to describe the murder within the first 24 h and the first year of life respectively (Pitt and Bale 1995).

Participants without a history of filicide and with ongoing postpartum mental illness (*N* = 30, thereafter *postpartum illness group*) were systematically and consecutively recruited from a state-funded perinatal psychiatry outpatient service. Only women who presented with postpartum severe mental illness were approached and included in the study. Postpartum severe mental illness was defined as a psychotic or affective disorder severe enough to require medical attention with onset within 6 months after delivery.

Postpartum healthy controls without a history of filicide (thereafter *control group*) were consecutively recruited among participants to antenatal courses at the same outpatient service used to recruit the postpartum illness group. Women were excluded from the control group if they had any known personal history of psychiatric disorders (*N* = 2) or reported family history of psychosis in first-degree relatives (*N* = 1).

All control participants and those in the postpartum illness group were 18 or older, had provided written informed consent, and had had a full-term delivery.

Clinical assessment included a face-to-face interview by a trained psychologist (AB) with the Structured Clinical Interview for DSM-IV Axis I Disorders (SCID-I) and the Structured Clinical Interview for DSM-IV Axis II Disorders (SCID-II) (First et al. 1997). Information on socio-economic, medical, and personal factors was collected using the clinical interview for DSM-IV (First et al. 1997). A clinical vignette and OPCRIT ratings were completed for all participants (McGuffin et al. 1991). Consensus best-estimate diagnoses were made according to DSM-V criteria (American Psychiatric Association 2013).

The analyses were conducted on anonymized data. Inference was conducted using the Fisher’s exact test (Fisher 1992) for categorical variables and the Mann–Whitney *U* rank test (Fay and Proschan 2010) for continuous variables.

## Results

### Characteristics of women who committed filicide

Five women committed neonaticide (i.e., filicide within 24 h of giving birth) and 11 infanticide (median 5 months after delivery). Fourteen filicides occurred between 13 and 96 months after delivery (thereafter “late filicide group,” median: 48 months after delivery).

Psychiatric diagnoses at the time of the filicide and lifetime are listed in Table 1**,** family history of psychiatric disorders, and psycho-social factors in Table 2**.**

**Table 1:** DSM-5 diagnosis at the moment of the filicide (acute diagnosis) and lifetime diagnosis. Participants could have more than one diagnosis

	Neonaticide (*N* = 5)		Infanticide (*N* = 11)		Late filicide (*N* = 14)	
	*N*	%	*N*	%	*N*	%
Acute diagnosis						
* No acute diagnosis*	*2*	*40%*				
* Schizophrenia spectrum and other psychotic disorders*			*2*	*18%*	*5*	*36%*
Schizophrenia; current psychotic major depressive episode			1	*9%*		
Brief psychotic disorder with peripartum onset					1	*7%*
Schizo-affective disorder; manic type; multiple episodes, currently in acute episode						
Schizo-affective disorder; depressive type; multiple episodes, currently in acute episode			1	*9%*	3	*21%*
Delusional disorder; multiple episodes, currently in acute episode					1	*7%*
* Bipolar and related disorders*			*5*	*45%*	*5*	*36%*
Bipolar I disorder						
Major depressive episode						
* With mood-congruent psychotic features*					2	*14%*
Bipolar II disorder						
Major depressive episode						
* With mixed features*			1	*9%*	1	*7%*
* With peripartum onset*			3	*27%*		
* With mood-congruent psychotic features*			5	*45%*	2	*14%*
* Depressive disorders*			*4*	*36%*	*4*	*29%*
Major depressive episode						
* With mood-congruent psychotic features*			4	*36%*	4	*29%*
* Dissociative disorders*	*3*	*60%*	*3*	*27%*		
Depersonalization-derealization disorder			2	*18%*		
Dissociative amnesia	1	20%	1	*9%*		
Dissociative identity disorder	2	40%				
* Substance-related and addictive disorders*	*1*	*20%*	*2*	*18%*	*1*	*7%*
* Personality disorders*	*4*	*80%*	*5*	*45%*	*6*	*43%*
Borderline personality disorder	3	60%	1	*9%*	3	*21%*
Paranoid personality disorder			3	*27%*	1	*7%*
Narcissistic personality disorder	1	20%				
OCD personality disorder			1	*9%*	2	*14%*
Psychiatric history						
* Previous psychiatric history*	3	60%	5	*45%*	13	*93%*
* Lifetime diagnosis (if different from current episode)*						
Major depressive disorder	1	20%				
Bipolar II disorder	1	20%				
Insomnia disorder			1	*9%*		
* Psychopathology during pregnancy*	1	20%	8	*73%*	4	*29%*
* Psychopathology with onset within 4 weeks after childbirth (only in late filicide group)*						
Psychotic depression or agitated depression or mixed episode					6	*43%*
Hypomania followed or following depression					2	*14%*
Depression without psychosis					4	*29%*

Italics refer to the different levels of specification—the diagnoses are like a tree with branches. For example: for schizophrenia spectrum and other psychotic disorders, the totals are 2 (18%) for infanticide and 5 (36%) for late infanticide. If we then break these numbers by specific diagnosis, we have the following: 1 schizophrenia: current psychotic major depressive episode and so on

**Table 2:** Demographic, clinical and psycho-social characteristics of the 90 women in the study

	Filicide (*N* = 30)		Postpartum illness (*N* = 30)		Healthy controls (*N* = 30)	
Age at interview (years, median and range)	34.5	22–44	35	25–52	33.5	27–43
Timing of the interview (months postpartum, median and range)	10	0–96	8.5	1–25	8	1–60
Diagnoses	*N*	%	*N*	%	*N*	%
Schizophrenia spectrum and other psychotic disorders	7	23%	2	7%		
Bipolar and related disorders	10	33%	2	7%		
Depressive disorders	8	27%	21	70%		
Posttraumatic stress disorder	0	0%	7	23%		
Obsessive compulsive disorder	0	0%	2	7%		
Anxiety disorders	0	0%	3	10%		
Dissociative disorders	6	20%	1	3%		
Substance-related and addictive disorders	4	13%	1	3%		
Personality disorders	15	50%	21	70%	5	17%
Psychotic symptoms	*25*	*83%*	*11*	*37%*		
Severe insomnia	*16*	*53%*	*3*	*10%*		
Thoughts of harming the baby	*3*	*10%*	*7*	*23%*		
Previous psychiatric history	*21*	*70%*	*26*	*87%*		
Socio-economic and clinical factors						
Age at delivery (years, median and range)	31.5	19–40	34	25–51	32	26–42
Primiparae (*N*, %)	14	47%	25	83%	23	77%
Years in education (median, range)	13	8–18	13	8–18	13	8–18
Low socio-economic background (*N*, %)	11	37%	7	23%	3	10%
History of childhood sexual abuse (*N*,%)	10	33%	11	37%	2	7%
History of adult sexual abuse (*N*, %)	2	7%	4	13%	3	10%
Family history of bipolar disorder or schizophrenia spectrum disorders (*N*, %)	15	50%	11	37%	NA	
Family history of violent death (*N*, %)	8	27%	1	3%	0	0%

Italics refer to the different levels of specification—the diagnoses are like a tree with branches. For example: for schizophrenia spectrum and other psychotic disorders, the totals are 2 (18%) for infanticide and 5 (36%) for late infanticide. If we then break these numbers by specific diagnosis, we have the following: 1 schizophrenia: current psychotic major depressive episode and so on

In the *neonaticide subgroup*, three women suffered a dissociative episode. Two of them had a previous history of psychiatric illness; one had suffered an episode of major depression during pregnancy. For the two remaining women in the group, there was no evidence of any acute psychiatric illness at the time of the neonaticide, nor prior to the event. Both were however diagnosed with borderline personality disorder at the SCID-2 interview. All women came from a lower socio-economic background and did not have a degree. Four of them had less than 8 years of education. None of the five women was receiving any psychiatric care at the time of the neonaticide.

All women in the *infanticide subgroup* met the diagnostic criteria for a major depressive episode at the time of the event, two in the context of a lifetime psychotic disorder, five for bipolar II disorder, and four for unipolar depression. Positive psychotic symptoms were always present. Three women also met the diagnostic criteria for dissociative disorders. In two women, hypomanic symptoms not meeting the diagnostic criteria for hypomania preceded the depressive episode. Eight out of 11 women had suffered a psychiatric episode during pregnancy—six a major depressive episode and two psychosis. Only one woman had a history of psychosis prior to the pregnancy and for only one woman there had been previous concerns over child safeguard.

At the time of the infanticide, no woman was taking antipsychotics nor mood stabilizers. Four women were taking selective serotonin reuptake inhibitors in monotherapy (three sertraline, one in combination with zolpidem, one escitalopram), including one with a previous history of bipolar disorder and two who had hypomanic episodes later in life, after the infanticide. Five women were not receiving any care and were not in contact with services, despite a previous history of severe psychiatric disorders.

Most women came from a higher socio-economic background. Women were represented across all education levels.

Like the *infanticide subgroup*, all women in the *late filicide subgroup* suffered depressive symptoms at the time of the event. For two women, psychotic symptoms were prominent. All women, except one with mixed depressive and manic features, experienced positive psychotic symptoms together with depression. All women had a previous psychiatric history, including 13 who had suffered a perinatal episode: 11 women had postpartum depression, three with mixed features, and five with psychosis. In two cases, depression was either following or followed by hypomanic symptoms. One woman experienced depression during pregnancy but was well after delivery.

Only 5 women were receiving specialist psychiatric care, and only three were taking antipsychotic/mood stabilizing treatment at the time of the filicide. Four women, all with a previous history of psychosis or bipolar disorder, were taking selective serotonin reuptake inhibitors in monotherapy (either sertraline or citalopram). The other three women were taking one haloperidol monotherapy, one haloperidol and mirtazapine, and one olanzapine and zolpidem.

Twelve out of fourteen women had a family history of psychosis or bipolar disorder in first- or second-degree relatives.

Strikingly, eight women (27%, five in the late filicide, two in the infanticide, and one in the neonaticide subgroup) had a family history of violent death, defined as either suicide or homicide up to third-degree relatives.

### Comparisons between women with severe postpartum mental illness and healthy controls

The median time at the interview of controls and women in the postpartum illness group was similar (8 and 8.5 weeks respectively).

Comparing clinical characteristics of women who committed filicide with those with severe postpartum mental illness who did not, different patterns emerged. Women who committed filicide were more likely to suffer psychotic symptoms (OR 8.3; CI 95% 2.26–36.13), severe insomnia (9.8; 2.28–61.75), and a schizophrenia spectrum or bipolar diathesis (4.8; 1.22–23.86). Rates of women with personality disorders, thoughts of harming the baby, and previous psychiatric disorders did not statistically differ between the two groups.

Although no woman in the healthy control group reported a psychiatric diagnosis, one in six met the DSM criteria for a personality disorder. The proportion of women who committed filicide from low socio-economic status was significantly higher than that of controls (5.0; 1.13–32.12). The sample size was too small to detect smaller differences between the filicide group and women with a history of severe postpartum mental illness. Stratified analyses of the three filicide subgroups (i.e., neonaticide, infanticide, and late filicide) were also not possible because of the small sample size. The association between low socio-economic status however seemed to be driven by women with a history of neonaticide.

Rates of history of sexual abuse in childhood were higher in both the filicide and the severe postpartum mental illness groups compared to controls (6.7; 1.25–70.46 and 7.8; 1.47; 80.47 respectively). Conversely, we did not observe any difference in the rates of history of sexual abuse in adulthood across groups.

## Discussion

To the best of our knowledge, this is the first clinical case–control study comparing the clinical and socio-demographic characteristics of women who committed filicide with both screened healthy controls and women with postpartum mental illness.

As previously reported (Friedman et al. 2005a; Resnick 1970; Spinelli 2001), the clinical picture of women with a history of neonaticide differed from that of women with a history of infanticide and late filicide. Four of the five women with a history of neonaticide had a personality disorder, and two of them did not meet the diagnostic criteria for any DSM acute postpartum illness. On the contrary, psychosis and depression were the most prevalent clinical features in cases of infanticide and late filicide.

The socio-economic background and level of education also differed, with women in the neonaticide group more likely to come from a disadvantaged background than the other two filicide groups.

When compared to women with postpartum mental illness, the most striking associations with filicide were family history of violent death and sub-optimal/absent psychiatric care in the postpartum period and at the time of the filicide. As most women in the filicide group had previous psychiatric history, our results underscore the importance of appropriate and prompt care.

In many cases of filicide, the mother was treated with selective serotonin (or serotonin and noradrenaline) reuptake inhibitor (SSRI and SNRI) monotherapy despite the bipolar/psychotic diathesis of her illness. The use of SSRI or SNRI monotherapy in women with a known history of bipolar disorder or psychotic disorders is controversial. The International Society for Bipolar Disorders (ISBD) task force report on antidepressant use in bipolar disorders explicitly advises against antidepressant monotherapy in bipolar I disorder (Pacchiarotti et al. 2013). Evidence from a national registry study suggests that antidepressant monotherapy in bipolar disorder carries an almost three-fold risk of incident mania (Viktorin et al. 2014). Our data therefore suggest that either the bipolar/psychotic diathesis was not recognized or that antidepressants were used despite the evidence of possible harm and lack of efficacy. Our results replicate the findings of a retrospective case note review study conducted in Korea on 45 women committing or attempting filicide (Kim et al. 2008). In the study, only one in three cases of bipolar diathesis was recognized at the admission, with over 60% of women with unipolar depression later diagnosed with bipolar disorder. Misdiagnosis of bipolar disorder in the postpartum period is common (Sharma et al. 2008). Even when the bipolar diathesis is not clear from the anamnesis or current presentation, psychiatric episodes in the immediate postpartum are potential markers of a bipolar diathesis, with an over fourfold relative risk of conversion to bipolar disorder—14% in a 15-year follow-up period in a Danish registry study (Munk-Olsen et al. 2012).

Most of the “lessons learnt” from the UK and Ireland Confidential Enquiries into Maternal Deaths and Morbidity 2017–2019 (MBRRACE-UK) apply to our study. (1) communication between health care professionals and coordinated care is fundamental. (2) Immediate risk factors should be identified by assessing psychiatric history, escalating symptoms, including psychosis and lack of sleep, and any associated behavior. Other red flags for suicide (i.e., suicidality, self-harm, and incompetency as a mother or estrangement) should also be assessed. (3) Women with a history of bipolar disorder or schizophrenia spectrum and other psychotic disorders should receive individualized risk assessment and close monitoring. Bipolar I disorder carries a 1 in 5 risk of postpartum mania or psychotic depression (Di Florio et al. 2013), compared to 1–2 in 1000 in the general population (Yang et al. 2022). Women may have been well for many years and not be in contact with psychiatric services and still may have a psychotic relapse in pregnancy or, more likely, after childbirth. For this reason, it is paramount that all health care professionals involved in the care of women in the perinatal period, even outside psychiatry, receive continuing education and training and learn to identify women at risk. Engaged family members are an important asset; however, women may not have the social support they need in their family or circle of friends. In any case, family members “should not be given responsibilities beyond their capabilities or be expected to act as a substitute for an effective mental health response” (MBRRACE-UK). (4) Over 10% of women in the filicide group had substance-related and addictive disorders. Substance disorders are a marker of vulnerability and put women at greater risk of postpartum relapse, even in the case of an apparent improvement in pregnancy. Women with substance-related disorders require engagement with the services across the entire perinatal period (MBRRACE-UK).

Although the sample size was too small to detect small effects and draw robust conclusions, we did not find any evidence of an association between infanticide/late filicide and personality disorders. Similarly, there was no specific association between filicide and sexual abuse in adulthood or low socio-economic background. On the contrary, we found a strong association with less prevalent risk factors such as family history of violent death and sexual abuse in childhood. While previous studies had linked maternal filicide to maternal history of abuse (Friedman et al. 2005b; Korbin 1986; Lewis and Bunce 2003; Mugavin 2008; Stanton et al. 2000), our research provides to the best of our knowledge the first evidence of an association between history of violent death in the family and filicide.

### Methodological considerations

Our findings require replication in larger samples. Given that filicide is an extremely rare outcome, large clinical studies that systematically investigate its risk factors and the clinical characteristics are very difficult to conduct. They would require a long-lasting, major financial and research effort to recruit and carefully assess an adequate number of cases (and matched controls). Our paper underscores the importance of forensic research of systematic, structured face-to-face assessment of women who commit filicide. Had our study been limited to case note reviews, we would have missed some cases of personality disorders and classified them as unipolar depression cases of bipolar disorder. Similarly, the choice of a diverse range of recruitment settings allowed us to better capture the complex psychopathology underpinning filicide.

For example, compared to previous studies that were limited to criminal justice and forensic psychiatry settings, our study included also participants with a history of filicide who were living in the community. The high prevalence of mental illness in our sample of women who committed filicide may reflect an overrepresentation of women recruited in psychiatric rather than criminal justice settings.

## Conclusion

Filicide is the rare and tragic outcome of a complex, heterogenous chain of vulnerabilities and events. Family history of violent death and childhood sexual abuse represent relevant antecedents. The lack of adequate postpartum psychiatric care is an important precipitating factor in many cases of infanticide and even late filicide. Perinatal care represents an opportunity for improvement and prevention. Our study underscores the need for increasing awareness by health care professionals and the wider society of the complex dynamics and psychiatric risks associated with motherhood.
